# Comparative Analysis of Endodontic ISO Size 06, 08, and 10 Stainless Steel K-Files Used for Glide Path Procedures

**DOI:** 10.3390/dj12040098

**Published:** 2024-04-10

**Authors:** Abayomi Omokeji Baruwa, Filipa Chasqueira, Sofia Arantes-Oliveira, João Caramês, Duarte Marques, Jaime Portugal, Jorge N. R. Martins

**Affiliations:** 1 Faculdade de Medicina Dentária, Universidade de Lisboa, 1600-277 Lisboa, Portugal; baruwaabayomi@gmail.com (A.O.B.); sofiaaol@campus.ul.pt (S.A.-O.); carames@campus.ul.pt (J.C.); duarte.marques@campus.ul.pt (D.M.); jaimeportugal@edu.ulisboa.pt (J.P.); 2 Instituto Universitário Egas Moniz, 2829-511 Monte da Caparica, Portugal; filipach@gmail.com; 3 Grupo de Investigação em Materiais Dentários (BIOMAT), Unidade de Investigação em Ciências Orais e Biomédicas (UICOB), 1600-277 Lisboa, Portugal; 4 LIBPhys-FCT UID/FIS/04559/2013 (https://doi.org/10.54499/UIDB/04559/2020), 1600-277 Lisboa, Portugal; 5 Instituto de Implantologia, 1070-064 Lisboa, Portugal; 6 Grupo de Investigação em Bioquimica e Biologia Oral (GIBBO), Unidade de Investigação em Ciências Orais e Biomédicas (UICOB), 1600-277 Lisboa, Portugal; 7 Centro de Estudos de Medicina Dentária Baseada na Evidência (CEMDBE), 1600-277 Lisboa, Portugal

**Keywords:** buckling, endodontics, glide path, microhardness, root canal therapy, stainless steel

## Abstract

Small-sized stainless steel hand files are conventionally employed in root canal treatment procedures for canal scouting and for glide path establishment, owing to their superior flexibility and proficiency in navigating confined spaces. Given the diversity of brands available in the market, there exists potential variability in their physical characteristics, thereby influencing clinical performance. Consequently, this study aims to conduct a comparative analysis of the design, metallurgy, and mechanical characteristics among seven stainless steel hand file brands across ISO sizes 06, 08, and 10. A total of 315 new 25 mm length stainless steel hand files with apical sizes of 0.06, 0.08, and 0.10 from seven distinct brands were included in the study. A meticulous inspection of all instruments was undertaken to identify any structural deformations that might render them ineligible for the study. The design inspection involved the random selection of instruments from each group, which were examined under various microscopes, including a dental operating microscope, optical microscope, and scanning electron microscope. Furthermore, two instruments from each group underwent energy-dispersive X-ray spectroscopy analysis for elemental composition documentation. Mechanical tests were conducted to evaluate the instruments’ resistance to lateral deformation (buckling) and their microhardness. Statistical analysis was executed using the nonparametric Mood’s median test, with a predetermined significance level of 0.05. Regarding the instruments design, all files exhibited an active blade length ranging from 16 to 17 mm. However, variations were observed in the number of spirals, tip designs, and sizes, with the API K-File notably larger in sizes 0.06 and 0.08 compared to the other instruments. Despite uniform elements composition, differences in geometric features and mechanical properties were evident. Concerning buckling strength, the API K-File demonstrated superior performance across all tested sizes, while the Dentsply ReadySteel, SybronEndo, and Mani K-Files exhibited lower results (*p* < 0.05). In microhardness assessments, both the API and Oro K-Files displayed the lowest outcomes, with medians of 531 HVN and 532 HVN, respectively, whereas the SybronEndo K-File exhibited the highest microhardness (657 HVN). Despite similar metallurgical composition, the observed distinctions in geometric features and mechanical properties underscore the impact of the manufacturing process on the characteristics of glide path stainless steel endodontic files. These disparities may ultimately influence their clinical performance.

## 1. Introduction

Mechanical instrumentation is fundamental in the field of endodontics, playing a pivotal role in determining the success and long-term prognosis of root canal treatments. This process involves the use of specialized instruments to clean, shape, and disinfect the intricate root canal system, aiming to eradicate microbial pathogens and prevent re-infection. The significance of mechanical instrumentation cannot be overstated, as it profoundly influences the overall quality and durability of endodontic procedures. A critical aspect of this process is the thorough removal of pulp tissue, debris, and bacteria from the root canal space, essential for eliminating potential sources of infection and reducing the risk of post-treatment complications. Mechanical instrumentation enables the precise shaping of the canal, facilitating effective irrigation and disinfection throughout the entire root canal system. This is particularly crucial in complex anatomies, where manual techniques alone may fall short in achieving comprehensive cleanliness. Furthermore, proper mechanical instrumentation aids in establishing an ideal shape for obturation with biocompatible materials, which is vital for preventing bacterial ingress and ensuring the long-term success of the root canal treatment. Advancements in technology, such as mechanized instruments, have further refined mechanical instrumentation, offering increased precision and efficiency. These innovations allow for a more controlled and predictable shaping of the root canal, ultimately improving the overall quality of endodontic treatments. Therefore, the importance of mechanical instrumentation in root canal treatments lies in its ability to thoroughly clean, shape, and disinfect the root canal system, contributing to a successful prognosis by reducing the risk of infections and creating an optimal environment for obturation [1].

The scouting and exploration of the root canals, after access cavity procedures, is a crucial step in the root canal systems’ biomechanical preparation during endodontic procedures. Small-diameter stainless steel hand files are the preferred tools for this preliminary exploration, allowing for the effective establishment and maintenance of a glide path in preparation for the subsequent canal enlargement steps [2,3]. According to Kwak et al. [4], the creation of a glide path is essential to reduce errors during canal preparation, particularly in areas with narrow and uneven lumens. Historically, small-diameter stainless steel hand files, typically ranging from sizes 0.06 to 0.10, have been employed for scouting due to their inherent flexibility and ability to navigate through constricted spaces [5]. While small files are recommended for coronal pre-flaring to ensure minimal contact with dentin walls and straight-line access to the apical terminus before transitioning to larger files [5,6], their use poses a significant challenge due to susceptibility to buckling forces, as demonstrated in previous studies [4]. This challenge becomes particularly relevant when navigating through calcified canals, where the intrinsic fragility of these smaller files becomes a concern. The inadequate resistance to buckling pressures can act as a barrier to achieving apical advancement, especially in anatomically challenging situations such as calcified root canals. The risk of applying excessive force in these circumstances is significant and may lead to unfavorable outcomes, such as the formation of ledges, deviations, or, in severe cases, file breaking [4,6]. To mitigate potential risks associated with the inherent fragility of these small files, practitioners must exercise caution and precision when using them. Balancing the necessity for rapid canal exploration with the delicate nature of the instruments employed is crucial for a successful and complication-free endodontic procedure. Therefore, practitioners must be aware of the possible problems posed by small files to negotiate complexities noted during root canal procedures with the utmost care and competence.

To mitigate potential errors, it is recommended to follow a systematic approach when using glide path files, progressively moving from ISO size 06 to 10 while employing gentle clockwise and counter-clockwise watch-winding motions adapted to specific clinical circumstances. However, in addition to the clinician’s skill and technique, the mechanical and physical attributes of these files significantly impact their overall performance. Previous research has affirmed the variability in properties among different glide path files [4,6,7,8,9,10]. Despite being constructed from similar alloys, each manufacturer has made alterations to their design to enhance the files’ rigidity, flexibility, and resistance to torsion, all with the goal of improving their efficiency in clinical practice.

Since the successful integration of mechanized nickel–titanium files in endodontics, replacing traditional manual root canal instrumentation, a growing trend has emerged. This trend involves replacing stainless steel manual glide path files with mechanized nickel–titanium ones, driven by their user-friendliness, preservation of canal shape, and a reduced occurrence of postoperative pain [7,11]. However, stainless steel files remain commonly employed for scouting and establishing the initial glide path before using the mechanized ones [4]. Unlike extensively studied nickel–titanium files, there are limited studies exploring the mechanical and physical characteristics of these stainless steel glide path files. Therefore, the primary objective of this study was to conduct a comparative analysis of seven different brands of stainless steel hand K-files (ISO sizes 06, 08, and 10), focusing on their geometric design, metallurgical properties, microhardness, and buckling strength. The null hypothesis to be tested was that there would be no differences in the mechanical performance between instruments from different commercial brands.

## 2. Materials and Methods

A global sample comprising 315 newly manufactured stainless steel K-files, ranging in apical sizes of 0.06, 0.08, and 0.10, each with a length of 25 mm, was gathered from seven distinct manufacturers (Dentsply ReadySteel K-File [Dentsply, Ballaigues, Switzerland], SybronEndo K-Files [Sybron Endo, Orange, CA, USA], Mani K-Files [Mani, Tochigi, Japan], API K-Files [Nilratan Tradelink Limited, New Dehli, India], Oro K-Files [Oro Dental, New Dehli, India], Dentsply Lexicon C-Files [Dentsply], and Dentsply ReadySteel C-File+ [Dentsply]). For metallurgical and mechanical assessments, a total of 15 endodontic instruments per group were included. Prior to testing, all files were inspected microscopically (Opmi Pico, Carl Zeiss Surgical, Jena, Germany) at a 13.6× magnification to identify major deformations, such as unwinding, which would exclude them from the investigation. It is noteworthy that no instruments were deemed unsuitable and subsequently excluded during this preliminary inspection stage.

### 2.1. Geometric Design Inspection

A total of 6 instruments per group underwent a comprehensive visual inspection using an endodontic operating microscope (Opmi Pico) at a magnification of 13.6× to determine the active area length of their blades and the number of cutting spirals. Subsequently, the files underwent microscopic analysis utilizing a conventional scanning electron microscope (Hitachi S-2400, Hitachi, Tokyo, Japan) (SEM). This analysis included recording specific parameters such as tip geometric design (200×), the configuration of symmetrical or asymmetrical cutting spirals (40×), and the identification of surface irregularities (300×). In the final stage of evaluation, the files were embedded in acrylic, transversely sectioned using a metal cutter, and meticulously inspected using a laboratory optical microscope (Meiji Saitama, Tokyo, Japan) with a magnification of 10× to assess their cross-section design.

### 2.2. Metallurgical Assessment

To characterize the elemental composition of the metal wire components, two files from each group underwent screening using energy-dispersive X-ray spectroscopy (EDS). The assessment was conducted on a conventional scanning electron microscope (Zeiss DSM 962, Carl Zeiss Microscopy GmbH, Munich, Germany) equipped with appropriate EDS detectors. Prior to assessment, the files were cleaned in a 2 min acetone bath to guarantee a superior material cleanliness. Subsequently, the tested files were positioned in a specimen support and stabilized within the scanning electron microscope vacuum chamber. The vacuum was maintained for 300 s, and the assessment settings included a 3.1-ampere filament current, 20-kilowatt acceleration voltage, and 25 mm work distance. The semi-quantitative analysis of the metal elements composition was carried out using an Inca x-act EDS detector (Oxford Instruments NanoAnalysis, Abingdon, UK) with data obtained from backscattered electrons. Data acquisitions were conducted with a 1 min lifetime, accepting an approximated 30% death time. All tests were performed on a file active area of 400 µm × 400 µm utilizing number 5 processing time. ZAF correction was applied, and the final outcomes were evaluated using The Microanalysis Suite V4.14 (Oxford Instruments NanoAnalysis, Abingdon, UK) software. The recorded atomic percentages of iron, chromium, and nickel were recorded.

### 2.3. Buckling Performance

The assessment of buckling performance was conducted using a universal testing machine (Instron Corporation 4502; series no H3307, Bucks, UK) equipped with a 1-kilonewton load cell. To determine the most appropriate sample size, a calculation was performed based on the results of the initial five tested instruments, focusing on groups showing the most significant differences. Considering an alpha of 0.05, a power of 80%, and an effect size with standard deviation of 0.88 ± 0.51 (0.06 files; Dentsply ReadySteel K-File vs. API K-Files), 0.94 ± 0.52 (0.08 files; Mani K-Files vs. API K-Files), and 0.58 ± 0.35 (0.10 files; Mani K-Files vs. API K-Files), sample sizes of 7, 6, and 7 files were determined, respectively. To account for the groups with intermediate results, in the initial trial, which was not considered in this calculation, a final sample size of 10 files was established. The instruments to be tested were positioned perpendicular to the floor plane, with their grip secured to the testing machine head and the tip pointing downward onto a small slot carved on the stainless steel testing base [10]. The assessment involved applying compressive stress at a rate of 1 mm per 60 s in the axial up–down direction of the instrument until a lateral displacement of 1 mm was observed. The maximum buckling load was measured in Newtons (N).

### 2.4. Microhardness Assessment

The microhardness assessment was carried out using a Vickers hardness tester (Duramin; Struers Inc., Cleveland, OH, USA). For the evaluation of microhardness, the 0.10 K-files were considered representative of the metal wire for each manufacturer and, consequently, only these files underwent microhardness testing. Each 0.10 instrument underwent five Vickers indentations. The adequate sample size was determined considering the groups that exhibited the most significant differences after an initial five indentations. Assuming an 80% power and an alpha of 0.05, and based on an effect size and standard deviation of 183.6 ± 102.9 (0.10 files; Oro K-Files vs. Dentsply Lexicon C-Files), a sample size of 7 indentations was determined. Considering that not all groups were used for this calculation, a final sample size of 15 indentations (3 instruments in total with 5 indentations each) was established. Preparation of specimens for this test followed the American Standards for Testing Materials (ASTM) recommendations [12]. Each tested instrument was mounted in an acrylic specimen holder and stabilized to be marked by the diamond penetrator using a 100 g/force (gf) pressure load for 15 s [13]. The indentation marks were inspected using a magnification objective of 40×. The microhardness outcomes were measured in hardness Vickers number (HVN).

### 2.5. Statistical Analysis

The buckling and microhardness results were presented with both means and standard deviations, as well as medians and interquartile ranges. The normality of the outcomes was evaluated using the Shapiro–Wilk test. As the results indicated a non-Gaussian distribution, the nonparametric Mood’s median test was employed to compare the groups of instruments in both mechanical assessments. The significance level was set at 0.05 (SPSS v.28.0.0.0 [190] for Windows; IBM SPSS Statistics, Chicago, IL, USA).

## 3. Results

**Figure 1:**
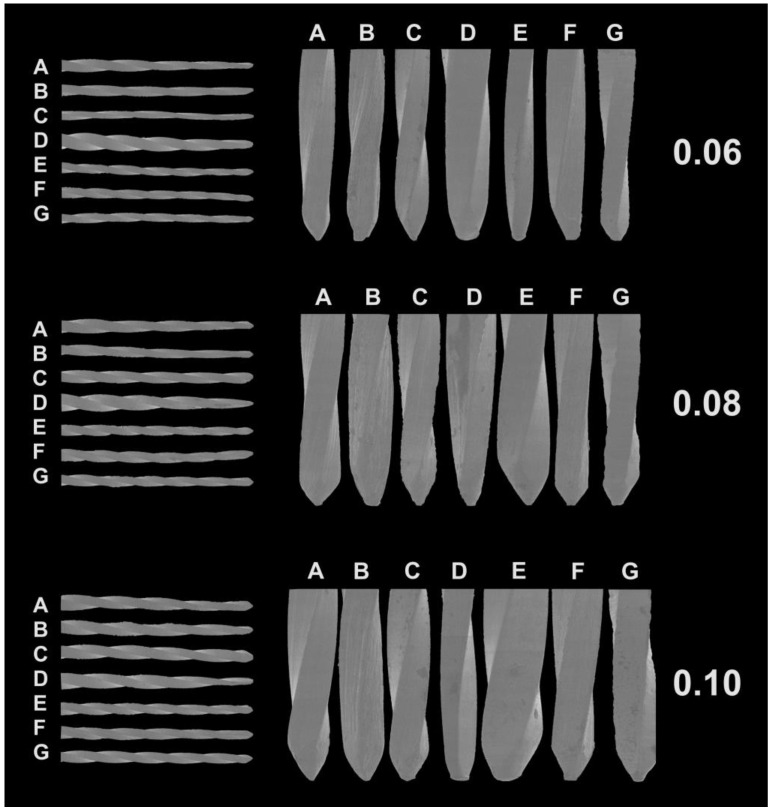
Scanning electron microscope representative images of the instruments’ cuttings spirals (**left**) and tip designs (**right**). Symmetrical spiral designs and distinct files tips could be noted. ([A] Dentsply ReadySteel K-File; [B] SybronEndo K-File; [C] Mani K-File; [D] API K-File; [E] Oro K-File; [F] Dentsply Lexicon C-File; and [G] Dentsply ReadySteel C-File+.)

The current study provides a comprehensive examination of various stainless steel hand files, offering valuable insights into their mechanical and physical characteristics. The analyzed features encompassed the active blade length, ranging between 16 and 17 mm, and the number of spirals, varying from 24 (Dentsply ReadySteel C-File+) to 42 (SybronEndo K-File) (Table 1). Notably, the SybronEndo K-File exhibited the highest spiral density (2.47 spirals/mm) while the Dentsply ReadySteel C-File+ displayed the lowest density (1.50 spirals/mm). A noteworthy observation was the design uniformity observed among the 0.06, 0.08, and 0.10 groups within the same commercial brands (Table 1). Microscopic inspection revealed symmetrical spiral geometries with diverse tip designs for all instruments (Figure 1). Particularly, the API K-File showed a notably larger size in the 0.06 and 0.08 instruments (Figure 1).

**Figure 2:**
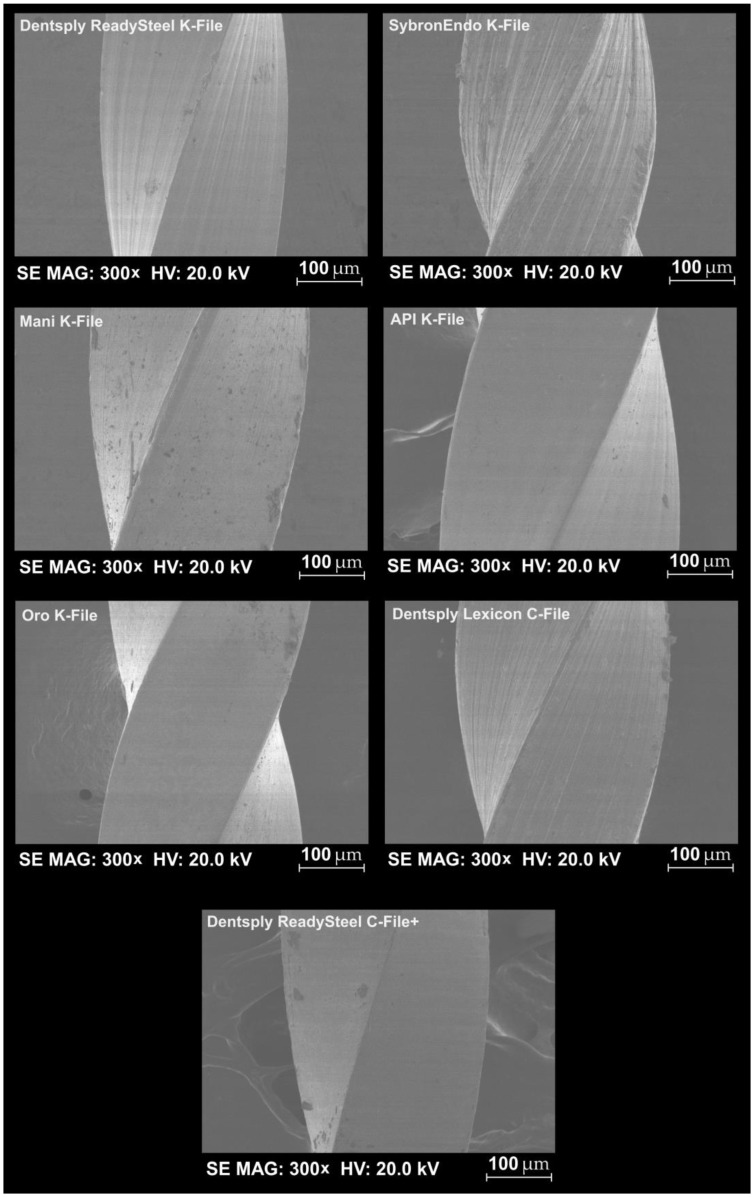
Scanning electron microscope representative images of the 0.10 instruments surface. The files API K-File, Oro K-File, and Dentsply ReadySteel C-File+ showed the least irregularities.

**Figure 3:**
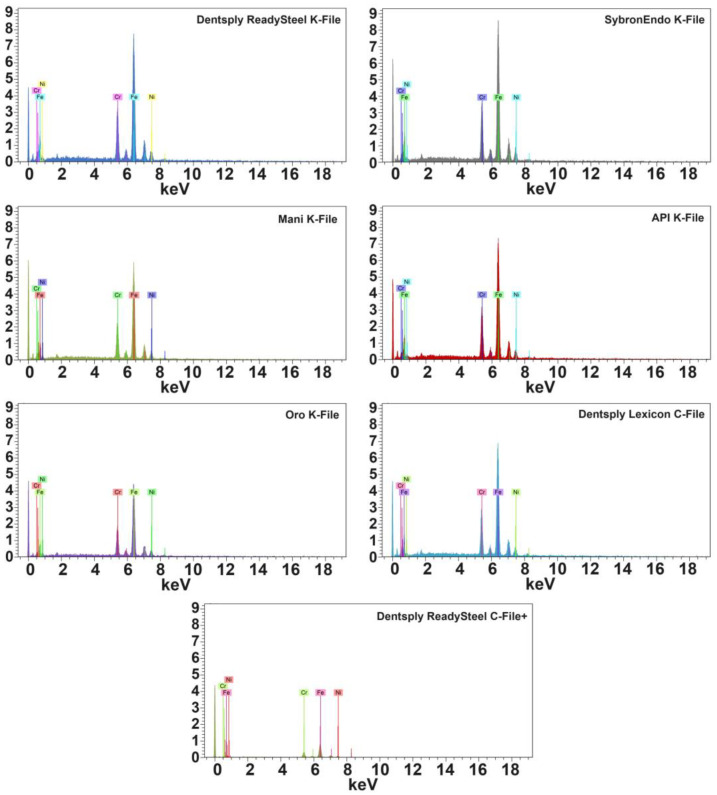
The EDS assessment representative 0.10 K-files spectrometers confirmed the stainless steel nature of the files’ metal wires.

**Figure 4:**
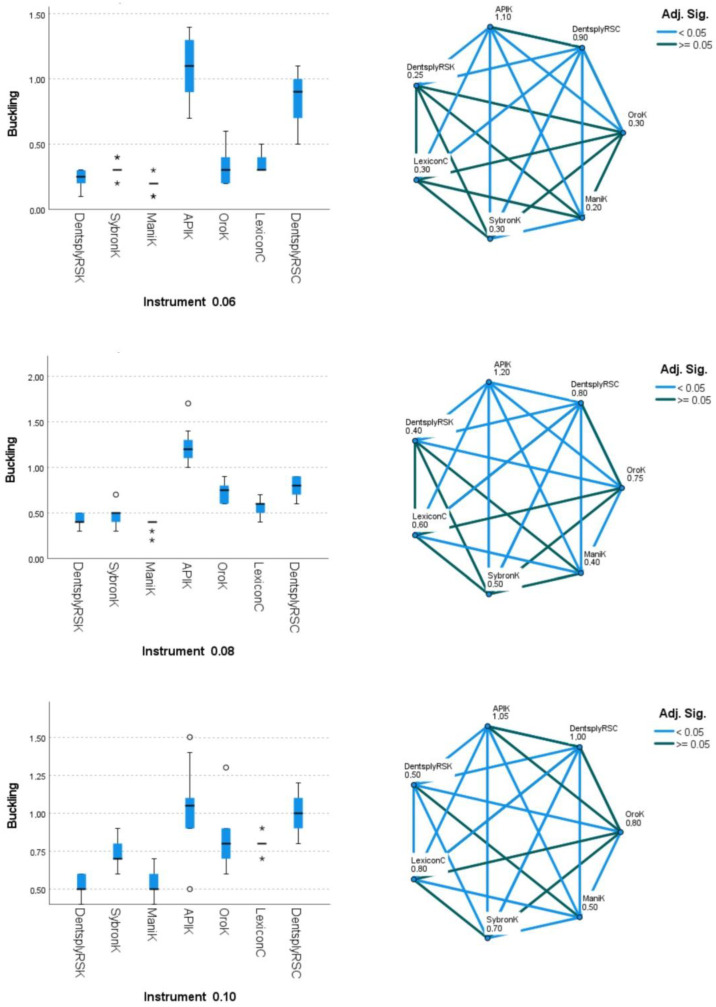
Buckling strength (medians) for all tested files. The box and whisker plots (**left**) show the buckling outcomes, while the pairwise comparisons (**right**) depict the differences between groups (DentsplyRSK: Dentsply ReadySteel K-File; SybronK: SybronEndo K-File; ManiK: Mani K-File; APIK: API K-File; OroK: Oro K-File; LexiconC: Dentsply Lexicon C-File; and Dentsply RSC: Dentsply ReadySteel C-File+).

**Figure 5:**
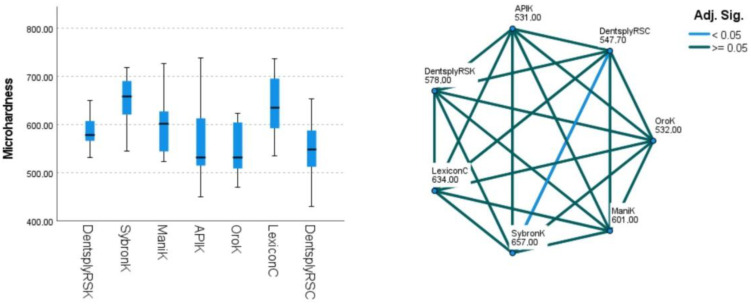
Microhardness assessment outcomes (medians) for the 0.10 K-file instruments. The box and whisker plots (**left**) depict the microhardness results, while the pairwise comparisons (**right**) show the differences between groups (DentsplyRSK: Dentsply ReadySteel K-File; SybronK: SybronEndo K-File; ManiK: Mani K-File; APIK: API K-File; OroK: Oro K-File; LexiconC: Dentsply Lexicon C-File; and Dentsply RSC: Dentsply ReadySteel C-File+).

The API K-File, Oro K-File, and Dentsply ReadySteel C-File+ groups exhibited minimal surface imperfections, indicative of smoother manufacturing processes (Figure 2). Additionally, all groups displayed a consistent square cross-section geometry, demonstrating uniformity in this aspect (Table 1). The energy-dispersive X-ray spectroscopy (EDS) test confirmed that all files were constructed from stainless steel metal wires, with equivalent atomic proportions of iron, chromium, and nickel observed across groups (Figure 3 and Table 2). Regarding the mechanical properties, the 0.06 (median 1.10 N), 0.08 (1.20 N), and 0.10 (1.05 N) file groups showed maximum buckling strength in the API K-File. Conversely, the buckling strength values for Dentsply ReadySteel K-File, SybronEndo K-File, and Mani K-File were lower (*p* < 0.05) (Figure 4 and Table 1). In terms of microhardness evaluation, the results look well balanced, with few significant differences between file systems. SybronEndo K-File displayed the highest microhardness (median of 657 HVN), whereas API K-File and Oro K-File had the lowest results (with median values of 531 and 532 HVN, respectively) (Figure 5 and Table 3).

## 4. Discussion

The concept of a glide path is pivotal in endodontics, with the objective of establishing a consistently smooth and reproducible pathway from the canal orifice to the apical terminus. Small stainless steel K-files are frequently employed for this purpose, facilitating a secure transition to larger files in subsequent procedures [5]. Given the frequent exposure of these small files to bending and torsional stresses, it becomes essential to investigate their physical and compositional characteristics to comprehend their adaptability in specific clinical circumstances.

Numerous factors, including cross-sectional shape, cross-sectional area, tip diameter, taper of the instruments, and manufacturing techniques, have been documented as influential in determining the mechanical properties of endodontic instruments [7,11,14,15]. The qualitative assessment of the present instruments, with sizes 0.06, 0.08, and 0.10, revealed similarities in their cross-sectional shape. Moreover, they demonstrated an almost uniform active blade length, ranging between 16 mm and 17 mm, with a relatively equal distribution of metallurgical composition. However, a notable distinction emerged in the tip design, particularly in the case of the API K-File, which exhibited a notably larger size compared to the other instruments. This difference became more pronounced when examining the 0.06 and 0.08 instrument sizes.

The influence of instrument size becomes prominently evident when the results are examined in relation to mechanical strength test outcomes [16]. Notably, the API K-Files demonstrated the highest buckling strength alongside the second-lowest Vickers hardness value. These findings potentially hold implications for both the tensile strength and cutting efficiency of these endodontic instruments [15,17]. This aligns with the concept that instruments with a greater metal mass generally showcase improved torsional and buckling strength, factors that can significantly influence the procedures involved in negotiating orifices and establishing a path to the apical area of the root canal [4].

Conversely, in comparison to other files, the Dentsply ReadySteel K-File, SybronEndo K-File, and Mani K-Files demonstrated reduced resistance to buckling pressures across all sizes, these findings corroborating those of a previous study conducted on similar brands of ISO size 15 stainless steel K-files [15]. This suggests that these particular instruments possess a higher degree of flexibility, a critical attribute for their performance in endodontic procedures. As previously highlighted by Allen et al. [9], the number of spirals in a file directly influences its flexibility, with an increasing number enhancing flexibility but potentially compromising cutting efficiency and overall rigidity—a pivotal factor in the successful outcome of root canal instrumentation. 

The heightened flexibility of these files offers specific advantages, particularly in negotiating the inherent curvature of the apical canal. This feature is especially valuable during the scouting phase, minimizing the risk of introducing abnormalities. However, it is important to recognize that increased flexibility and reduced susceptibility to buckling, while advantageous in many scenarios, may pose challenges in fully navigating restricted and calcified root canals. The greater flexibility may also lead to significant plastic deformation, diminishing the overall effectiveness. In contrast, C+ files, characterized by the fewest spirals, exhibit a higher level of rigidity [10,15]. This enhanced stiffness translates into greater cutting efficiency, enabling a more assertive approach to tissue removal [8]. While this heightened aggressiveness improves procedural speed and efficacy, it raises concerns about an increased likelihood of iatrogenic errors, such as transportation and ledging [18]. Striking a delicate balance between flexibility and stiffness is paramount, underscoring the necessity for practitioners to meticulously select and adapt their instrument choices based on the specific features of each clinical case, ensuring the effectiveness and safety of root canal treatments.

Research indicates a growing prevalence of nickel–titanium glide path files in modern endodontics [4,11,19]. Their widespread adoption is attributed to reported advantages, notably in reducing apical extruded debris. This reduction has been associated with decreased postoperative pain and minimized canal transportation when compared to traditional K-files, suggesting that the use of nickel–titanium files for glide path preparation leads to improved overall preparation outcomes [20,21,22]. Additionally, the manual creation of a glide path is recognized as a time-consuming, technique-specific process that may result in less than ideal preparation. Consequently, nickel–titanium glide path files are emerging as the preferred choice for this critical phase [20,23]. However, despite their efficacy, challenges arise in certain root canals, with irregularities in the apical third and complex canal morphology, particularly when scouting the apical 1 to 3 mm using solo nickel–titanium glide path files [5]. As a result, in such intricate cases, the usage of manual stainless steel glide path files becomes necessary. 

In the present study, we conducted a comprehensive analysis of seven distinct commercial brands of stainless steel hand K-files in sizes 0.06, 0.08, and 0.10. Our observations revealed noteworthy variations in geometric designs and overall mechanical performance among these files. Specifically, API and Oro K-Files exhibited less consistent results compared to their counterparts. Despite the uniform material composition of stainless steel, the observed discrepancies can be attributed to purposeful slight variations in metallurgical compositions and the intricacies of the manufacturing process [15,24]. This indicates that these glide path files are not interchangeable and may not perform uniformly in similar clinical situations. It is therefore critical for practitioners to carefully evaluate and understand the distinctive characteristics of every glide path file. The subtle modifications, which may have a major influence on their efficacy in various clinical circumstances, are highlighted by the diversity in geometric designs and mechanical performance. Making informed decisions about the use of stainless steel hand files in endodontic instrumentation requires an understanding of these distinctions. By doing so, professionals may enhance the accuracy and efficacy of root canal therapy, ensuring the best possible results even in challenging anatomical circumstances [25].

One strength of the present research lies in its utilization of a diverse range of evaluation techniques and methodologies to conduct an in-depth analysis of glide path files. This approach provides deeper insights into these endodontic tools than previous studies have been able to offer. Additionally, the inclusion of a large number of brands and the assessment of multiple instrument sizes contribute to understanding characteristic patterns within the entire set of smaller files for a particular commercial brand. However, it is important to acknowledge the limitations of this study. One such limitation is the assessment of only a specific number of brands, leaving out others that may warrant proper investigation. Another limitation is the relatively restricted number of mechanical tests conducted. Results from torsional and bending tests, or assessments of cutting ability, would be valuable and could provide further clarification on certain aspects. Recognizing these limitations and incorporating a wider variety of files and tests would be a viable direction for future investigations. Increasing the study’s scope will improve the scientific knowledge of glide path files holistically.

## 5. Conclusions

From this study, it was possible to conclude that variations exist in the mechanical performance of different brands of stainless steel hand K-files. Specifically, API K-File demonstrated the highest buckling strength across all sizes (0.06, 0.08, and 0.10), while SybronEndo K-File exhibited the highest microhardness. These findings suggest that the manufacturing process plays a significant role in shaping the properties of these smaller glide path files. Therefore, the clinical choice of files should be guided by consideration of their specific properties and the clinical case requirements.

## Figures and Tables

**Table 1:** The design characteristics of endodontic files and buckling results are presented as mean (±standard deviation) and median [interquartile range].

Instrument	Type	Design				
		Active Area Length	Number of Spirals	Number of Spirals/mm	Cross-Section	Buckling (Newton)
0.06 Files						
Dentsply ReadySteel K-File	K-file	16 mm	32	2.00	Square	0.24 (±0.07); 0.25 [0.20–0.30]
SybronEndo K-File	K-file	17 mm	42	2.47	Square	0.31 (±0.06); 0.30 [0.30–0.33]
Mani K-File	K-file	17 mm	34	2.00	Square	0.19 (±0.06); 0.20 [0.18–0.20]
API K-File	K-file	17 mm	32	1.88	Square	1.11 (±0.23); 1.10 [0.90–1.32]
Oro K-File	K-file	17 mm	32	1.88	Square	0.32 (±0.12); 0.30 [0.20–0.40]
Dentsply Lexicon C-File	C-file	17 mm	30	1.76	Square	0.34 (±0.07); 0.30 [0.30–0.40]
Dentsply ReadySteel C-File+	C-file	16 mm	24	1.50	Square	0.84 (±0.21); 0.90 [0.65–1.00]
0.08 Files						
Dentsply ReadySteel K-File	K-file	16 mm	32	2.00	Square	0.41 (±0.07); 0.40 [0.38–0.50]
SybronEndo K-File	K-file	17 mm	42	2.47	Square	0.47 (±0.11); 0.50 [0.40–0.50]
Mani K-File	K-file	17 mm	34	2.00	Square	0.37 (±0.07); 0.40 [0.38–0.40]
API K-File	K-file	17 mm	32	1.88	Square	1.24 (±0.20); 1.20 [1.10–1.33]
Oro K-File	K-file	17 mm	32	1.88	Square	0.74 (±0.12); 0.75 [0.60–0.83]
Dentsply Lexicon C-File	C-file	17 mm	30	1.76	Square	0.56 (±0.11); 0.60 [0.48–0.63]
Dentsply ReadySteel C-File+	C-file	16 mm	24	1.50	Square	0.79 (±0.10); 0.80 [0.70–0.90]
0.10 Files						
Dentsply ReadySteel K-File	K-file	16 mm	32	2.00	Square	0.53 (±0.07); 0.50 [0.50–0.60]
SybronEndo K-File	K-file	17 mm	42	2.47	Square	0.72 (±0.09); 0.70 [0.68–0.80]
Mani K-File	K-file	17 mm	34	2.00	Square	0.54 (±0.08); 0.50 [0.50–0.60]
API K-File	K-file	17 mm	32	1.88	Square	1.05 (±0.28); 1.05 [0.90–1.18]
Oro K-File	K-file	17 mm	32	1.88	Square	0.82 (±0.20); 0.80 [0.68–0.90]
Dentsply Lexicon C-File	C-file	17 mm	30	1.76	Square	0.80 (±0.05); 0.80 [0.80–0.80]
Dentsply ReadySteel C-File+	C-file	16 mm	24	1.50	Square	0.99 (±0.12); 1.00 [0.90–1.10]

**Table 2:** Metal wire characteristics of the tested endodontic instruments.

Instrument	Type	Metal Wire	Atomic Percentage		
			Iron	Chromium	Nickel
0.06 Files					
Dentsply ReadySteel K-File	K-file	Stainless steel	74.48	17.98	7.54
SybronEndo K-File	K-file	Stainless steel	73.89	17.78	8.33
Mani K-File	K-file	Stainless steel	74.68	17.74	7.58
API K-File	K-file	Stainless steel	74.58	18.28	7.13
Oro K-File	K-file	Stainless steel	74.33	17.79	7.88
Dentsply Lexicon C-File	C-file	Stainless steel	74.35	17.99	7.66
Dentsply ReadySteel C-File+	C-file	Stainless steel	75.03	18.69	6.28
0.08 Files					
Dentsply ReadySteel K-File	K-file	Stainless steel	75.12	16.89	7.99
SybronEndo K-File	K-file	Stainless steel	74.49	16.69	8.83
Mani K-File	K-file	Stainless steel	75.31	16.65	17.74
API K-File	K-file	Stainless steel	75.26	17.18	7.56
Oro K-File	K-file	Stainless steel	74.95	16.70	8.35
Dentsply Lexicon C-File	C-file	Stainless steel	74.98	16.89	8.12
Dentsply ReadySteel C-File+	C-file	Stainless steel	75.76	17.57	6.66
0.10 Files					
Dentsply ReadySteel K-File	K-file	Stainless steel	75.21	16.85	7.94
SybronEndo K-File	K-file	Stainless steel	74.62	16.73	8.65
Mani K-File	K-file	Stainless steel	74.12	17.27	8.61
API K-File	K-file	Stainless steel	75.01	16.67	8.32
Oro K-File	K-file	Stainless steel	75.26	16.23	8.51
Dentsply Lexicon C-File	C-file	Stainless steel	74.53	17.28	8.18
Dentsply ReadySteel C-File+	C-file	Stainless steel	75.02	17.16	7.82

**Table 3:** Microhardness results (shown as mean (±standard deviation); median [interquartile range]).

Instrument	Type	Microhardness (Hardness Vickers Number)
0.10 Files		
Dentsply ReadySteel K-File	K-file	586 (±32); 578 [566–607]
SybronEndo K-File	K-file	649 (±54); 657 [598–704]
Mani K-File	K-file	598 (±61); 601 [542–628]
API K-File	K-file	561 (±77); 531 [513–615]
Oro K-File	K-file	547 (±52); 532 [508–605]
Dentsply Lexicon C-File	C-file	638 (±65); 634 [590–712]
Dentsply ReadySteel C-File+	C-file	549 (±61); 547 [508–592]

## Data Availability

Data are contained within the article.
